# A retrospective review of spinal masses at a South African tertiary neurosurgery unit in Durban (2020–2024)

**DOI:** 10.4102/jcmsa.v4i1.306

**Published:** 2026-03-13

**Authors:** Nikesh Deveduthras, Thalia Govender, Rivona Harricharan, Naseera Omar

**Affiliations:** 1 Discipline of Neurosurgery, Faculty of Surgery, University of KwaZulu-Natal, Durban, South Africa; 2 Department of Medicine, Faculty of Surgery, University of KwaZulu-Natal, Durban, South Africa

**Keywords:** spinal masses, schwannoma, meningioma, laminectomy, primary tumours, secondary tumours, ependymoma

## Abstract

**Background:**

The prevalence of spinal masses is not well documented in South Africa. Understanding their occurrence, distribution and histopathological spectrum is crucial for optimising diagnosis, treatment, and management strategies in clinical practice.

**Methods:**

A retrospective, descriptive analysis was conducted for patients who underwent spinal surgery for spinal masses between 01 January 2020, and 31 December 2024. Descriptive statistics, chi-square tests, and correlation analyses were used to assess relationships between demographics, mass type, surgical intervention, and outcomes.

**Results:**

Twenty-eight patients were included in the study, with a mean age of 37.74 years (standard deviation [s.d.] 18.19). The most common tumours were schwannomas (28.6%) and meningiomas (21.4%). Laminectomy was the most common surgical procedure performed (60.7%), followed by laminoplasty (21.4%) and fusion (7.1%). A significant negative correlation was found between age and follow-up duration (*r* = −0.579, *p* = 0.001) and between age and tumour type (*r* = −0.568, *p* = 0.002). A significant positive correlation between surgical intervention and mass type (*r* = 0.541, *p* = 0.003) was also found.

**Conclusion:**

This study demonstrated that schwannomas and meningiomas were the most predominant types of spinal masses observed within the cohort. Laminectomy was the principal surgical approach utilised.

**Contribution:**

Significant associations between patient age, tumour type, and surgical interventions may inform future clinical decisions and research.

## Introduction

Spinal masses vary in prevalence, type, and demographic distribution across different regions. Although primary spinal tumours are relatively uncommon, comprising only around 3% of all central nervous system (CNS) tumours in adults, they hold significant clinical importance within neurosurgical practice because of their potential to cause profound neurological deficits and functional disability.^1,2^ Spinal tumours can produce sequelae such as neuropathic pain, pathological fractures, spinal instability, nerve root compression, and, in more severe cases, spinal cord compression.^2,3^ Spinal tumours in South Africa, both primary and secondary, are less commonly encountered compared to infectious spinal pathologies such as tuberculosis (TB) with metastatic tumours, predominantly lung adenocarcinoma, breast cancer, and prostate cancer being the most frequent.^4.5^

The differences in the occurrence of spinal masses are unclear but are likely influenced by demographic, environmental, and healthcare factors contributing towards the limited data on primary spinal tumours in South Africa. In addition, there are more comprehensive studies conducted by developed countries compared to developing countries making use of data registers.^6^ Recognising these variations is essential for developing targeted strategies to improve diagnosis, management, and resource distribution for spinal diseases both locally and internationally.

The primary objective of this study is to review the spinal masses encountered at Inkosi Albert Luthuli Central Hospital in Durban, South Africa from 2020 to 2024 to provide insights into the clinical presentation, surgical trends, and outcomes for South African patients with spinal masses. Furthermore, the study aims to compare local occurrences with international and explore potential reasons for any differences.

## Research methods and design

### Study design and setting

A retrospective, descriptive analysis was conducted within the Neurosurgery Department at Inkosi Albert Luthuli Central Hospital in Durban, South Africa between 01 January 2020 and 31 December 2024.

### Inclusion and exclusion criteria

Male and female patients of all ages and patients who underwent surgical intervention for intradural, extradural, or intramedullary spinal cord masses with histopathological confirmation were included in this study. Exclusion criteria comprised of patients with isolated degenerative spine disease, trauma patients as well as patients without a confirmed spinal mass reported on magnetic resonance imaging (MRI). Patients with known or suspected TB of the spine or primary bone tumours are managed by a nearby orthopaedic spine unit and were excluded from this study. An exception involved the TB abscess, which was not suspected initially because of being reported as a spinal mass, but diagnosed later on histology and microbiology analysis.

### Data collection

Data were obtained from the hospital software system for clinical records (Sorian^TM^, Siemens, Germany) including patient demographics (age, sex), mass type (based on imaging report), surgical procedure, histopathological diagnosis, and follow-up duration.

### Statistical analysis

Stata V17 (Stata Corp, College Station, TX, United States) was used to analyse the data. Descriptive statistics were used to summarise the cohort characteristics (age and gender), tumour distribution (suspected lesion), surgical interventions (year operated, type of surgery performed and follow-up). Continuous variables were examined for normality and means (inclusive of standard deviation – s.d.) were used to represent parametric data. Non-parametric data were represented using medians (including interquartile ranges – IQR). Fischer’s tests were used to examine the associations between demographic and clinical factors including histology. Pearson correlation analysis was applied to explore the relationships between patient age, mass type, and surgical interventions. A *p*-value ≤ 0.05 was considered statistically significant.

### Ethical considerations

The study was reviewed and approved by the Biomedical Research Ethics Committee of the University of KwaZulu-Natal (BREC 00029720). This study was a retrospective review of existing medical records. As it involved the analysis of de-identified data collected during routine clinical care, no written or oral consent was obtained from participants. All data were handled in accordance with institutional and ethical guidelines. Patient identifiers were removed prior to analysis, and data were stored securely on password-protected computers accessible only to the research team.

## Results

**FIGURE 1:**
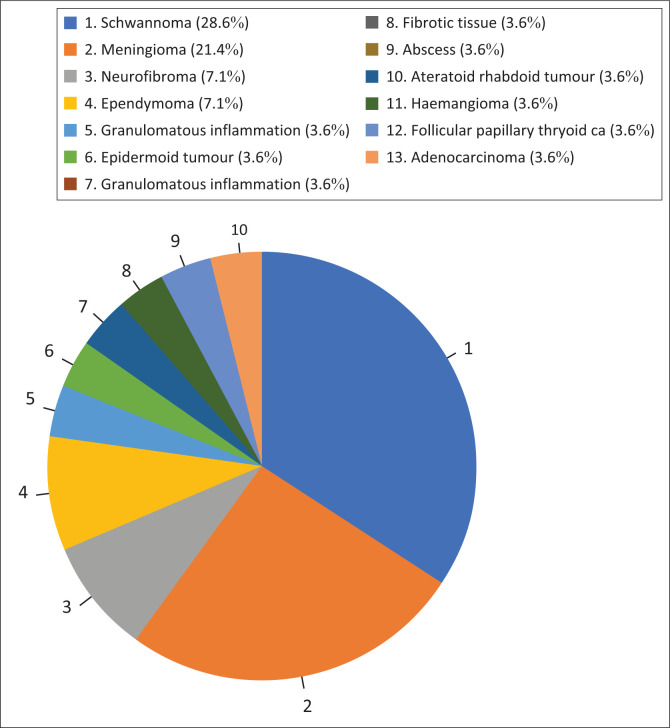
Histopathological distribution of spinal masses depicted in this chart.

A total of 28 patients who underwent spinal surgery for spinal masses were included in the study. The mean age of the cohort was 37.74 years (s.d. 18.19), with 16 female (57.1%) and 12 male (42.9%) patients. The year 2021 saw the highest frequency of surgeries (46.4%). Schwannomas were the most frequent tumour type, accounting for 28.6% of cases (see Table 1). Meningiomas were the second most common tumour type, comprising 21.4% of cases. Other tumour types included neurofibromas (7.1%), ependymomas (7.1%), and haemangiomas (3.6%). Less common diagnoses included epidermoid cysts, arachnoid cysts, follicular papillary thyroid carcinoma metastases, adenocarcinoma metastases, atypical teratoid and/or rhabdoid tumour (ATRT), granulomatous inflammation, fibrofatty tissue and abscesses each accounting for 3.6% of cases (Figure 1). Laminectomy was the most frequently performed surgical procedure, comprising 60.7% of all surgeries. Laminoplasty accounted for 21.4% of surgeries, while fusion was performed in 7.1% of cases. Pearson correlation analysis revealed a significant negative correlation between age and follow-up duration (*r* = −0.579, *p* = 0.001), as well as between age and suspected lesion type (*r* = −0.568, *p* = 0.002). A significant positive correlation was found between surgical intervention and suspected lesion type (*r* = 0.541, *p* = 0.003). The histopathological distribution of spinal masses is depicted in Figure 1.

**TABLE 1:** Histopathological distribution of spinal masses.

Histology result	*n*	%
Schwannoma	8	28.6
Meningioma	6	21.4
Neurofibroma	2	7.1
Ependymoma	2	7.1
Granulomatous inflammation	1	3.6
Epidermoid tumour	1	3.6
Ateratoid rhabdoid tumour	1	3.6
Haemangioma	1	3.6
Follicular papillary thryoid carcinoma	1	3.6
Adenocarcinoma	1	3.6
Inconclusive result	1	3.6
Granulomatous inflammation	1	3.6
Fibrotic tissue	1	3.6
Abscess	1	3.6

While age-specific and sex-specific tumour types were not found, the data analysis found that schwannomas, meningiomas and neurofibromas, total 11 (39.28%), were found predominantly in the 30 year to 50 year age group.

It was also found that most spinal masses in this cohort occurred in the 30 to 50 year age group, total 16, (57.14%), and the 0 to 29 year age group was the next largest group with a total of 7 (25%). There was also a female predominance of schwannoma, meningioma and neurofibromas, total 10, (35.71%) with a male predominance of ependymomas – total 2 (7.14%). The frequency of benign vs malignant tumours in this study is shown in Table 3.

**TABLE 2:** Distribution of spinal masses by location and type(non neoplastic).

Category	Frequency	%
Intradural extramedullary tumours	18	64.3
Intradural intramedullary tumours	3	10.7
Extradural and/or vertebral lesions	4	14.3
Infectious and/or inflammatory	2	7.2
Inconclusive	1	3.6

**Total**	**28**	**100**

**TABLE 3:** Classification of spinal masses as benign, malignant, or non-neoplastic according to histopathology.

Behaviour	Frequency	%
Benign	19	67.9
Malignant	3	10.7
Non-Neoplastic	5	17.9
Inconclusive	1	3.5

**Total**	**28**	**100.0**

In younger patients with age less than 30 years, there were mainly ependymoma, ATRT and epidermoid cyst found – total 3 (10.71%).

## Discussion

Spinal tumours have the potential to cause detrimental neurological consequences and their occurrence in South Africa is not well documented. In this study, we evaluated the occurrence of spinal masses that were operated at one major neurosurgical facility in the country between 2020 and 2024.

The median age of our study was 39.50 years (IQR 30.3–45.0) and the mean was 37.74. This was aligned to a recent large-scale study by Alvarez-Crespo et al., which reported the mean age of spinal schwannomas as 35.8 to 57.1.^7^ Often spinal meningiomas have a mean age of 60–69; however, this varies between individual studies. When assessing for gender disparities, there was a female predominance of 57.1%. This was in keeping with a global female predominance in some studies.^8^ However, this is contrasted by a male predominance in some countries such as Japan.^9^

In this study, spinal schwannomas (28.6%) and meningiomas (21.4%) were the most prevalent tumour types detected. These findings correlate with a study by Momin et al. where a higher incidence of meningiomas (21.4%) and a lower incidence of ependymomas (3.6%) were documented.^10^ According to a multicentre study by Hirano et al., schwannomas and meningiomas represent the two most frequently encountered intradural extramedullary tumours of the spine.^11^ Conversely, intradural intramedullary tumours such as astrocytomas and ependymomas are more prevalent in paediatric populations. At Korle-Bu Teaching Hospital, meningiomas were reported as the most frequent tumour type between 2007 and 2023.^1^ The predominance of schwannomas may be reflective of a higher incidence of peripheral nerve sheath tumours within our cohort; however more large-scale studies are warranted.

Understanding the aetiology of spinal masses allows for the appropriate course of management to be followed. Spinal tumour aetiology encompasses neoplastic and infectious causes. There is a link between advancing age and the probability of the tumour being metastatic. Previous studies support the notion that older age is associated with the risk of high-grade tumours and younger age with benign tumours.^2,5,12^ This study found a predominance of primary benign spinal tumours, however, it is important to note that both locally and internationally, metastatic or secondary spinal tumours, often arising from primary cancers such as those of the lung, breast, or prostate, are more frequently encountered than primary spinal tumours (Table 4).^4,9^ Less common were high-grade tumours that mimicked low-grade tumours on MRI. There was a predominance of intradural extramedullary tumours compared to intradural intramedullary tumours, which is in keeping with globally reported data (see Table 2).^1,10^ It was, however, in contrast to a study in China that found the primary malignant tumour was chordoma followed by ependymoma and primary benign tumours were haemangioma; however, the study looked at spinal bone tumours.^13,14^

**TABLE 4:** Distribution of primary and secondary spinal tumours in the study cohort.

Origin	Frequency	%
Primary Benign	19	86.4
Primary Malignant	1	3.5
Secondary	2	9.1

**Total**	**22**	**100.0**

Our study found that 18% of the masses encountered were non-neoplastic in nature, with granulomatous inflammation accounting for 3.6% of these cases. Although this percentage is relatively low, it remains clinically significant in our setting, particularly in HIV and/or TB-endemic regions, where infectious aetiologies must be carefully considered in the differential diagnosis.^4,5^ Spinal tuberculosis continues to be a major contributor to spinal disease in South Africa, largely attributable to the high burden of tuberculosis and HIV infection. Radiologically, spinal TB may closely resemble neoplastic processes, particularly in the presence of vertebral body destruction, paraspinal or epidural abscess formation, and spinal canal compromise. A 2013–2016 cohort from Groote Schuur Hospital found granuloma formation correlated with CD4 count (*r* = 0.503, *p* = 0.02), alongside vertebral collapse in HIV-positive patients with spinal tuberculosis.^15^

Laminectomy is a well-established surgical approach in the management of spinal tumours, providing access for tumour excision and decompression of neural structures. The most common surgical approach is posterior and via a laminectomy (60.7%) or laminoplasty (21.4%). This approach is likely favoured as it is less destructive of bony elements, causing a lower incidence of post-surgical spinal instability, as most commonly a single-level or two-level laminectomy is employed.^7,8^ A significant positive correlation was found between surgical intervention and suspected lesion type (*r* = 0.541, *p* = 0.003).

The findings of this study offer important insights into the spectrum of spinal masses encountered in Durban, KwaZulu-Natal, South Africa. Expanding knowledge of the causes of spinal masses aids clinicians in making informed decisions and optimising patient management.

### Limitations of the study

The study was limited to a single centre, and the time frame of the study and the sample size were small. Another factor that may have influenced the small sample size was the reduced number of surgical procedures performed in 2020 because of the coronavirus disease 2019 (COVID-19) pandemic and the restrictions. The higher surgical rate observed in 2021 may have been because of the rescheduled procedures. Future studies involving larger, multicentre cohorts are recommended to validate these findings and further explore regional variations. Furthermore, the difference in surgeons and their preferential surgical approach may be considered a limitation.

In addition, another limitation was inconsistent follow-up. Follow-up is dependent on numerous factors including age of the patient, level of education, availability of transport, financial resources, time constraints and willingness to participate. There should also be a focus on prospective studies examining long-term functional outcomes, genetic or environmental risk factors and strategies to promote earlier detection through public health initiatives. However, despite its limitations, this study provides one of the first documented accounts of spinal mass occurrence within a South African neurosurgical setting, offering important groundwork for future investigations.

## Conclusion

This retrospective study provides important insights into the occurrence and management of spinal masses at a major neurosurgery centre in Durban, South Africa. Schwannomas and meningiomas were the most encountered benign tumours, with laminectomy being the predominant surgical procedure. The correlations observed between patient age, mass type, and surgical intervention offer important implications for clinical practice and future research in spinal pathology locally. This study highlights the need for further research into spinal masses in South Africa, with the aim of improving diagnostic and management strategies. A potential next step could incorporate a multicentre study with a larger sample size, which evaluates spinal masses for an extended time frame and assesses the functional outcomes of patients.
